# Identification, Mapping, and Molecular Marker Development for *Rgsr8.1*: A New Quantitative Trait Locus Conferring Resistance to *Gibberella* Stalk Rot in Maize (*Zea mays* L.)

**DOI:** 10.3389/fpls.2017.01355

**Published:** 2017-08-03

**Authors:** Qian Chen, Jun Song, Wen-Ping Du, Li-Yuan Xu, Yun Jiang, Jie Zhang, Xiao-Li Xiang, Gui-Rong Yu

**Affiliations:** Institute of Biotechnology and Nuclear Technology, Sichuan Academy of Agricultural Sciences Chengdu, China

**Keywords:** maize stalk rot, next-generation sequence, QTL-seq, finely map, resistance QTL, *Gibberella*, candidate gene

## Abstract

Maize stalk rot is a major fungal disease worldwide, and is difficult to control by chemical methods. Therefore, in maize breeding, quantitative trait loci (QTLs) conferring resistance are important for controlling the disease. Next-generation sequencing technologies are considered a rapid and efficient method to establish the association of agronomic traits with molecular markers or candidate genes. In the present study, we employed QTL-seq, which is a whole-genome resequencing-based approach, to identify candidate genomic regions conferring resistance to maize stalk rot. A novel resistance QTL *Rgsr8.1* was finely mapped, conferring broad-spectrum resistance to *Gibberella* stalk rot (*GSR*). Segregation analysis in F_2_ and BC_1_F_1_ populations, which were derived from a cross between 18327 (Susceptible) and S72356 (Resistant), indicated that the resistance to *GSR* was likely to be a quantitatively inherited trait in maize. The result of QTL-seq showed that the resistance to *GSR* was mapped on chromosome 8 from 161.001 to 170.6 Mb. Based on the simple sequence repeat (SSR) markers, single-nucleotide polymorphism (SNP) markers, and the recombinant test, the location of *Rgsr8.1* was narrowed down to 2.04 Mb, flanked by SSR-65 and SNP-25 markers at the physical location from 164.69 to 166.72 Mb based on the maize reference genome. In this region, two candidate resistant genes were found with, one auxin-responsive elements and the other encoding a disease resistance protein. In summary, these results will be useful in maize breeding programs to improve the resistance to *GSR* in maize.

## Introduction

As one of the most devastating soil-borne diseases in maize (*Zea mays* L.), maize stalk rot occurs in all continents of the world (Francis and Burgess, 1975; Lal and Singh, 1984; Chambers, 1988; Ledencan et al., 2003; Cook, 2008). Maize stalk rot was firstly detected in China in the 1920s (Yang et al., 2002a), and has recently become a major threat to maize production. Furthermore, maize stalk rot also causes plant lodging and other issues, including yield reduction, low grain quality, and problems during harvest (Ledencan et al., 2003). White (1999) indicated that both fungal and bacterial pathogens can cause stalk rot in maize. *Fusarium graminearum* Schwabe (teleomorph *Gibberella zeae*) is one of the major stalk rot pathogens, causing *Gibberella* stalk rot (*GSR*) in maize, producing a wide variety of mycotoxins during pathogen invasion (Wu et al., 2007). Because of the soil-borne infection pathway, fungicides are ineffective in controlling *GSR*. Hence, the use of resistance gene(s) has been demonstrated to be both economical and the most effective method in controlling *GSR* (Yang et al., 2004, 2005, 2010).

Previous studies indicated that resistance to *GSR* was controlled by qualitative and quantitative genetic loci. Based on F_2:3_ families, deriving from the cross between “33-16” (susceptible line) and “B89” (resistant line), Pè et al. (1993) identified and mapped five *GSR* resistance quantitative trait loci (QTLs) on chromosomes 1, 3, 4, 5, and 10. In another study, a single dominant gene against *GSR* has been located with a confidence interval of 5 cM on chromosome 6 (Chen and Song, 1999; Yang et al., 2004). Another major resistance QTL, which is mapped on the long arm of chromosome 4, has been identified and cloned (Jung et al., 1994; Frey, 2005). Using simple sequence repeat (SSR) markers, Yang et al. (2004, 2005) mapped two *GSR* resistance genes on chromosomes 4 and 6. Based on the backcross population from the hybridization between the resistant line “1145” and the susceptible line “Y331,” Yang et al. (2010) reported that two QTLs were identified to confer resistance against *GSR*. Although recent studies have indicated that resistance to *GSR* is a quantitative trait and is controlled by multiple genes with additive effects, the specific inherited trait of resistance to *GSR* remains unclear. The symptom development of stalk rot depends on genetic factors, as well as environmental elements, such as soil moisture, climate change, and temperature (Parry et al., 1995). Several research studies have indicated that chemical application methods can decrease maize infections to the fungal pathogens (Ahmad et al., 1996; Dorn et al., 2009), but the identification and application of resistant genes may prove a more effective method in pathogen control.

Molecular mapping has been used for the identification of resistance genes. Moreover, it provides a possible starting point of gene cloning and marker-assisted selection in maize breeding (Foiada et al., 2015; Nair et al., 2015; Ku et al., 2016). However, the usual methods, conducted by genotyping segregating populations derived from bi-parental crosses, are time consuming and laborious (Salvi and Tuberosa, 2005). Bulked segregant analysis (BSA) has been considered a simplified approach to identify genes (Giovannoni et al., 1991; Michelmore et al., 1991). BSA technologies have identified and mapped important traits in many crops (Li et al., 2012; Trick et al., 2012). QTL-seq, a new technique combining next-generation sequencing (NGS) and BSA has been developed for gene mapping (Fekih et al., 2013; Takagi et al., 2013a,b, 2015). Research studies have used QTL-seq to identify genes in many crops, such as rice, wheat, and chickpea (Trick et al., 2012; Chen et al., 2015; Das et al., 2015; Xia et al., 2015; Zheng et al., 2016).

Previous attempts to map the resistance to *GSR* were always based on SSR, random amplified polymorphic DNA (RAPD) and restriction fragment length polymorphism (RFLP). To our knowledge, application of NGS technology to this aim has not been previously reported. In the present study, the QTL-seq approach was used to precisely localize the genomic region for *GSR* resistance. Using the classical analysis method, the single-nucleotide polymorphism (SNP) and SSR markers derived from the resistant genomic region were also used to finely map the major resistant QTL. The results from this study will be useful in breeding programs for improving maize resistance to *GSR*.

## Materials and Methods

### Plant Materials

Maize inbred lines “18237” (recurrent parent and highly susceptible to *GSR*, P1) and “S72365” (donor parent and completely resistant to *GSR*, P2) were crossed to produce the F_1_ hybrid, which was self-pollinated to generate the F_2_ population, and backcrossed with “18237” to generate the BC_1_F_1_ population. These two populations were grown at the experiment farm of the Sichuan Academy of Agricultural Sciences (Chengdu, Sichuan, China). Each individual was artificially inoculated with *F. graminearum*.

### Artificial Inoculation and Disease Evaluation for Symptoms

*Fusarium graminearum* was cultured on potato dextrose agar in darkness at 25°C for 4–5 days. The maize kernel was prepared by first dipping in water for 20 h at 37°C in darkness, then in boiling water for 1 h. The kernels were then dried on a ventilated table, and autoclaved for 20 min at 121°C within plastic bags. Preparing for field evaluation, the sterilized kernels were inoculated with *F. graminearum* at 25°C in complete darkness for 15 days. Field inoculation of plants was conducted as described by Yang et al. (2010).

Plants were evaluated for stalk rot symptoms twice a week, beginning 1 month post-inoculation. Typical symptoms of stalk rot were observed, such as browning reactions in lower internodes, spongy stem, wilting, lodging, and plant death. Evaluating mycelial growth and root damage requires the stem to be cut. Incidents of stalk rot infection was scored using a disease assessment scale of 1–9. Scales 1–2 were regarded as resistant and 8–9 were regarded as susceptible. Plants with a score of 9 were dead and lodging with broken vascular tissue of the stem; a score of 8 was similar to 9, the plant lodging down but with an unbroken stem; plants with a score of 7 exhibited withered leaves and a soft stem, but no lodging; a score of 6 corresponded to symptoms of withered leaves, but with a harder stem than in plants with a score of 7; plants with a score of 5 exhibited withered leaves, and a slightly soft stem; a score of 4 was assigned when parts of leaves were withered, and a normal stem was observed; a score of 3 was given for the observation of only leaf chlorosis; a score of 2 indicated some yellow leaves; and a score of 1 indicated no obvious symptoms.

### Illumina Sequencing and QTL-seq Analysis

**FIGURE 1:**
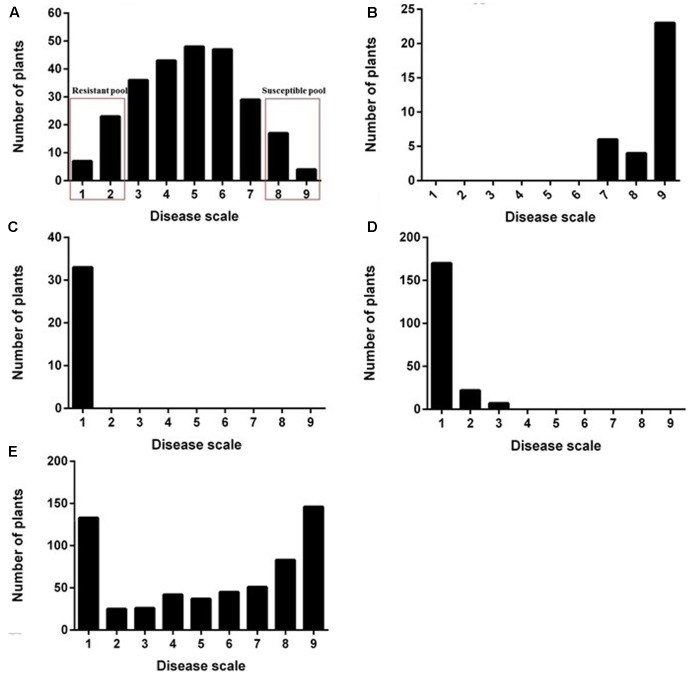
Distribution of disease severity for the two parental lines, F_1_ plants, F_2_ population, and BC_1_F_1_ population. The F_2_ population and the construction of R-pool and S-pool **(A)**. Susceptible parental line “18327” **(B)**. The resistant parental line “S72356” **(C)**. The F_1_ plants **(D)**. The BC_1_F_1_ population **(E)**.

DNA was extracted from fresh young leaves of single plants using the standard CTAB protocol (Doyle and Doyle, 1990). For QTL-seq, two DNA pools, susceptible pool (S-pool) and resistant pool (R-pool) were constructed, respectively, by mixing an equal amount of DNA from 25 F_2_ plants with the lowest disease scores and 25 F_2_ plants with highest disease scores (**Figure 1A**). A DNA concentration of 2–5 μg from each of the P1, P2, R-pool, and S-pool were used to construct pair-end sequencing libraries (150 bp read length, which were sequenced using Illumina HiSeq 2500 (Illumina Inc., San Diego, CA, United States) by Gene Denovo Biotechnology Co. (Guangzhou, China). Raw reads with >10% unidentified nucleotides and with >50% bases having phred quality scores of <20 were filtered out to get high-quality clean reads. To identify SNPs, these clean reads were mapped and aligned to the maize reference genome (RefGen_V4^1^) using the Burrows–Wheeler Aligner (BWA) software (Li and Durbin, 2009) with the settings as follow: mem 4 -k 32 -M. SNP-calling was performed for all samples using the SAM tools (Li and Durbin, 2009). The SNP positions with a read depth <6 and SNP-index <3 were filtered out. To confirm the physical positions of each SNP, the software ANNOVAR (Wang et al., 2010) was used to align and annotate SNPs.

In this study, the parameters of SNP-index and Δ (SNP-index) (Abe et al., 2012; Takagi et al., 2013a) were calculated to identify candidate regions for maize stalk rot resistant QTLs. The reference sequence for SNP-index calculation was developed by replacing the detected SNPs from one of the parental cultivars with those from the reference genome. The SNP-index represents frequencies of parental alleles in the population of pooled individuals. Slide window analyses with parameters “2 Mb windows size and 100 kb increment” was applied to SNP-index plots.

The Δ (SNP-index) was calculated based on subtraction of SNP-index between R-pool and S-pool. SNP-index is equal to “0” or “1” when entire reads contain genomic fragments from P1 or P2, respectively. The Δ (SNP-index) value will be significantly different from 0 in genomic regions with major QTL of the target gene (Takagi et al., 2013a). We calculated statistical confidence intervals of Δ (SNP-index) for all the SNP positions with given read depths under the null hypothesis of no QTLs, and plotted them along with Δ (SNP-index). With a 95% confidence interval in 10,000 bootstrap replicates, the Δ (SNP-index) was obtained for each read depth.

### Marker Development and QTL Analysis

To verify the major QTL for *GSR* resistance from QTL-seq, polymorphic markers were developed in the predicted region of maize chromosome 8. SSR markers in the predicted region were searched using the MISA tool^2^, and employed for polymorphism screening between two parental lines, and between the R-pool and S-pool. SNP markers that were in the predicted region of the QTL were converted to PCR-based markers, and the primers were designed using Primer 5^3^. Polymorphic markers were used to screen the F_2_ population. The linkage analysis was performed using the software JoinMap 4.1 (Van Ooijen, 2006) and recombination values were converted to centiMorgan (cM) using the Kosambi mapping function (Kosambi, 1943). The genetic information together with phenotyping data was used for QTL analysis using the composite interval mapping (CIM) model in WinQTL cartographer 2.5 software (Wang et al., 2012).

### Expression Analysis of the Candidate Genes for *GSR* Resistance

The expression of candidate genes was investigated using real-time quantitative PCR (qPCR). Leaf samples were collected from P1, P2, F_1_, F_2_-S (susceptible to *GSR*) and F_2_-R (resistant to *GSR*) individuals at the early stage of inoculated plants. Total RNA for all samples was extracted using Trizol Reagent (Invitrogen, Carlsbad, CA, United States) and digested with RNase-free DNase I (Takara Bio, Japan) for 30 min at 37°C. Reverse transcription was conducted by Super III Reverse Transcriptase (Invitrogen, San Diego, CA, United States). The qPCR primers for the candidate genes (Zm00001d011953 and Zm00001d011972) were 5′-CCAGCTGTACAGGAGCATGA-3′ (forward) and 5′-CCGGAACACGTCTTGGTAGT-3′ (reverse) for Zm00001d011953, 5′-AAAAGGCTTGTTGCTGGAGA-3′ (forward) and 5′-GGTGGAGGTGCATTTTGTCT-3′ for Zm00001d011972, respectively. qPCR was performed in a LightCycler^®^ 96 Real-time PCR Instrument (Roche, Swiss) with SYBR Green Real-time PCR Master Mix (Takara, Japan). The gene expression levels were determined using Ct value normalized with the formula 2^-ΔΔCt^ (Livak and Schmittgen, 2001). The maize *Actin* gene was employed as an endogenous control, with the following primers: 5′-GCCGGTTTCGCTGGTGATGATGCGCC-3′ (forward) and 5′-GTGATCTCCTTGCTCATACGATCGGC-3′. Three replicates were measured to calculate the average relative expression levels. A Student’s *t*-test was used to check the significant differences in expression levels among these five samples.

## Results

### Inheritance of *GSR* Resistance

Frequency distribution of resistance to *GSR* is presented in **Figures 1A–E**. Following artificial inoculation with *F. graminearum*, the inbred line “18327” (P1) plants showed severe stalk rot symptoms (**Figure 1B**); “S72356” (P2) exhibited complete resistance to *GSR* and no symptoms were observed (**Figure 1C**). Most of the F_1_ hybrids (85.3%) displayed high levels of resistance to *GSR* (**Figure 1D**), suggesting that the major *GSR* resistance allele might be dominant. The resistance to *GSR* in the F_2_ population showed continuous variation (**Figure 1A**), and a skewed distribution of disease severity was observed in BC_1_F_1_ population (**Figure 1E**). Based on these results, it was suggested that the resistance to *GSR* in P2 was likely to be a quantitatively inherited trait.

### Sequencing and QTL-seq Mapping

Based on library construction and NGS-based high-throughput sequencing of P1, P2 and two DNA-pools, a total of 344 Gb of data was generated, including 2.93 billion of 150 bp high-quality clean reads, and 98.44–98.56% high-quality reads were mapped on the reference genome. The average sequence depths were 20-fold in parents and 30-fold in pools. The total number of variants was 16, 997, 640, including 15, 490, 449 SNPs and 1, 507, 191 indels. The Q20 ratio ranged from 95.66 to 96.21% (**Table 1**).

**Table 1:** Summary of the sequencing results data.

Sample	Read length (bp)	Data generated (Gb)	High-quality clean reads	High-quality clean nucleotides (bp)	Alignment (%)	Q20 (%)	GC (%)
18237 (S)	150	62	402, 419, 030	59, 508, 707, 632	98.44	95.99	47.72
S72356 (R)	150	68	445, 375, 978	65, 037, 058, 791	98.54	96.20	47.89
Susceptible pool	150	102	673, 436, 682	98, 759, 887, 612	98.56	95.66	47.53
Resistant pool	150	112	746, 134, 292	109, 258, 655, 210	98.50	96.21	47.24


**FIGURE 2:**
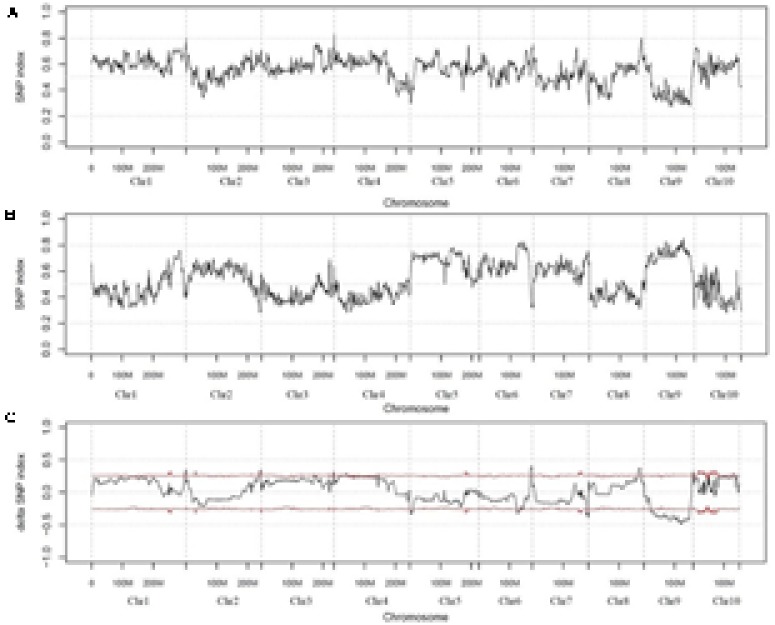
SNP-index graphs of R-pool **(A)**, S-pool **(B)**, and Δ (SNP-index) graph **(C)** from QTL-seq analysis. *X*-axis represents the position of 10 maize chromosome and *Y*-axis represents the SNP-index. A candidate QTL (*Rgsr8.1*) location was identified on maize chromosome 8 (161.004-170.535 Mb interval) with the criteria that the SNP-index in R-pool **(A)** was approximately 0.8, SNP-index in S-pool **(B)** was approximately 0.3, and the Δ (SNP-index) **(C)** was above the confidence value (*P* < 0.05).

To identify the candidate genomic region conferring resistance to *GSR*, the SNP-index was calculated, based on each SNP identified. The average SNP-index was calculated with a sliding window of 2 Mb intervals with 100 kb increment for S-pool and R-pool to detect the candidate genomic regions. SNP-index graphs were generated for R-pool (**Figure 2A**) and S-pool (**Figure 2B**) by plotting the average SNP-index against the position of each sliding window in the P1 genome assembly. It was expected that the SNP-index graphs of the R-pool and S-pool would be identical for the genomic regions that are not relevant to phenotypic difference, whereas the genomic region harboring the *GSR* resistance QTL should exhibit unequal contribution from P1 and P2 parental genomes. In addition, the SNP-index of predicted regions for R-pool and S-pool would appear as mirror images (Takagi et al., 2013a). After calculating and combining the information of SNP-index in R-pool and S-pool, Δ (SNP-index) was calculated and plotted against the genome positions (**Figure 2C**).

In the present study, the region of chromosome 8 ranging from 161.001 to 170.6 Mb had an average SNP-index higher than 0.60 in R-pool with the highest equal to 0.80. Conversely, the average SNP-index in the region of S-pool was lower than 0.45 with the lowest equal to 0.3. The predicted genomic region harboring the resistance QTL to *GSR* identified by QTL-seq were determined by Δ (SNP-index) value. The threshold of the Δ (SNP-index) value was 0.25 at the 95% significance level. Results showed that the genomic region on the chromosome 8 from 161.001 to 170.6 Mb was significantly different from 0 (**Figure 2C**). These data demonstrated that in maize, a major QTL conferring *GSR* resistance was present in the 161.001–170.6 Mb region on chromosome 8. We named this region as *Rgsr8.1*.

### Narrowing Down the Predicted Region by Polymorphism Markers

Based on 565 non-synonymous variations, a total of 45 SNP markers (Supplementary Table S1) were developed, which distributed equally over the predicted region according to physical position. A total of 729 SSR markers were searched from the predicted region. Among these SSR markers, 165 SSR markers (Supplementary Table S2), equally distributed on the predicted region, were used to analyze the polymorphism.

**FIGURE 3:**
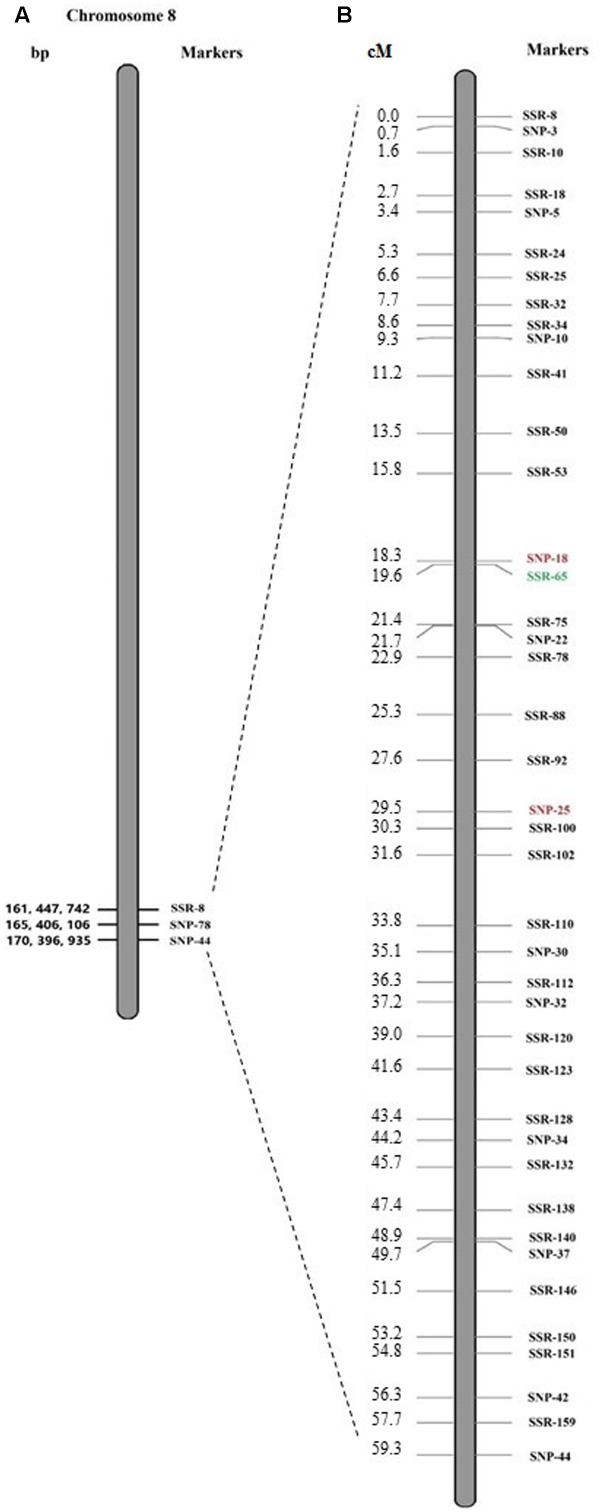
Integration of QTL-seq-predicted region on chromosome 8 **(A)** and traditional QTL mapping with SNP and SSR markers **(B)**. The genetic (cM) or physical (bp) positions, and the markers mapped on the chromosome are specified on the left and right side, respectively. The markers identified by QTL mapping and recombination test are marked in red and green, respectively.

**FIGURE 4:**
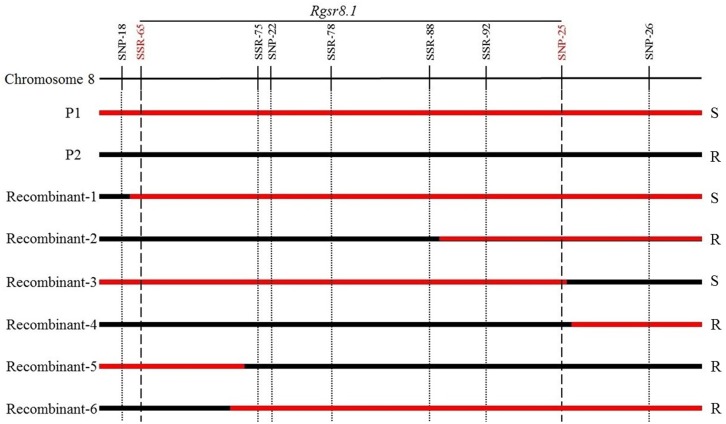
Examination of the recombinants in BC_1_F_1_ population using the polymorphism marker in **Table 4**. The *red bar* is the genome from 18327 (S). The *black bar* is the region from S72356 (R).

The 45 SNP markers and 165 SSR markers were checked for polymorphisms between P1 and P2, R-pool, and S-pool. Of the SNP markers, 33 markers amplified well, and 12 SNP markers were found polymorphic between P1 and P2, R-pool, and S-pool (**Table 2**). Twenty-nine SSR markers were identified to be polymorphic between P1 and P2, R-pool, and S-pool (**Table 3**). In total, 12 SNP markers and 29 SSR markers were used for QTL analysis based on the F_2_ populations. A major QTL for resistance to *GSR*, physically located in the region of 164.678–166.721 Mb on chromosome 8 (**Figure 3**), was flanked by two SNP markers (SNP-18 and SNP-25) with genetic distances of 4.57 and 6.62 cM. This result agreed with the QTL-seq analysis supporting a major *GSR* resistance QTL on chromosome 8. The LOD scores of the polymorphism markers within this region ranged from 0.26 to 45.23, and could explain 34.4% of the variance (**Table 4**). Additionally, we further narrowed down the *Rgsr8.1* locus by using recombination test, based on 6 BC_1_F_1_ recombinants, which were recovered within the region on chromosome 8. To figure out the physical position where the recombinant events occurred, eight markers (**Table 4**) were used to analyze the P1, P2, and recombinants. The results showed that no recombinants were detected except for SSR-65 and SNP-25. Therefore, the mapping data narrowed the *Rgsr8.1* locus down to a 2.04 Mb interval between the SSR-65 and SNP-25 (**Figure 4**). Furthermore, we used these eight markers to screen the F_2_ and BC_1_F_1_ population. We estimated that the SSR-78 marker was linked with *GSR* resistance in “S72356” via phenotypic and genotypic identification.

**Table 2:** The information of 12 SNP markers.

Loci	Forward primer (5′–3′)	Reverse primer (5′–3′)	Position (bp)
SNP-3	CGGAATATCTCGCAACAGGT	CTCTTCCTGGAGTCCTCGG	161, 466, 693
SNP-5	GTCATGGAGATGGAGGTCGT	ACGCTGCCTACCTCCGCT	162, 145, 869
SNP-10	GTCTTGGTTGGCATTCCACT	GTTTGAAAGCCCGTGGACTA	162, 949, 082
SNP-18	CGGTTACTACTACGGCAGCG	CAGTTGTAGTAGGACGCCCC	164, 677, 916
SNP-22	TTCCACCAGATCCTAAACGG	GCAGATGCTACCAAGGCTTC	165, 243, 672
SNP-25	CGTACCTCTTGACCTTGGGA	AGCTACCACGTGCTGTCCTT	166, 721, 266
SNP-30	CTGATGGCAGGGTTCAAAAT	AAAGGTGGCTTTGAGCTTGA	167, 474, 984
SNP-32	CCAACGCGTCGTTACAGTTA	CACTCACCTGCTCCTGCC	167, 839, 590
SNP-34	CCTAATAGTTTCCCCGGCTT	TATCTTCTCAGAGCAGCGCA	168, 610, 967
SNP-37	CCAAACCAATGCAACATCAG	TTGCCACGATATGGTCTTGA	169, 185, 032
SNP-42	TCAGCTCGCTCACATTTGTC	AACAATCTAGGATCGCGGAA	170, 084, 205
SNP-44	TGACAGGAGAGAATTTGGGG	CAAGCTCATTCCAAGCATCA	170, 396, 935


**Table 3:** The information of 29 SSR markers.

Loci	Forward primer (5′–3′)	Reverse primer (5′–3′)	Position (bp)
SSR-8	ATCTGTGGTGGTGTCACCTT	GAATTCACTGCTCCATGTGC	161, 447, 742
SSR-10	CATGAGGGCTGGATACTTGG	TTCGTTGGTACATTGATGTGG	161, 555, 503
SSR-18	CCCATGGGAAGTTGAACCTA	CAAGCCCCCTTATGATCTTG	162, 113, 979
SSR-24	ACCGTGATCTTTGGAAGTCG	GCATTCCGATAGGGATTACG	162, 415, 963
SSR-25	ATAGACGTCCGGATGTGGTC	AAGGCCTGATCACATAATCCA	162, 461, 145
SSR-32	CTGCAACTGAGATGGTCCAA	GGGTATCACGTCGTCTTCGT	162, 828, 637
SSR-34	TGTTTGGTTTGTGGAATGGA	CCGCTAAACTCGCACTTAGG	162, 924, 552
SSR-41	CAACTGGCTGTGCAAAGTGT	GACCCTTTCTGGATGGTTCA	163, 319, 000
SSR-50	AGCTTTTCACCTCCACGCTA	TAGCTCCAACACGTACACGG	163, 819, 019
SSR-53	AAGCCGATTCACTGAGCCTA	TTGTAGAGCTGCACCACGTC	163, 949, 635
SSR-65	AGCCGATGGACAAAAATTGA	TCGTCGTCTTCTGGACCTCT	164, 685, 091
SSR-75	GCTGGGAAGAGGAAGAGGTT	AAACAAGACGGGAACAAACG	165, 241, 204
SSR-78	ACACAAGAGGTGGGACAAGC	TGTACGTCTGGACCCTCTCC	165, 406, 106
SSR-88	CCAAGGCACAAGAAGAGAGC	GCATGCATGGAAGAGGTACA	166, 097, 976
SSR-92	AAAGACCAGTGGCGTTTAGC	GGCTCGGATGAGTCTGAGTT	166, 322, 914
SSR-100	GCACCTATATGAAGCCCAGG	CCCCAAACTTCCAAAAAGTG	166, 780, 249
SSR-102	AGTGAGCCTTGAGCACCATAG	AATTTCCATTGATTCGGTGC	166, 862, 760
SSR-110	CACCTATGCGCAGAGTTTGA	GGCATCGTTTTCTTTTCCAA	167, 308, 770
SSR-112	GCTCTGCTTCTCACTAGCGG	ACAGAGCCTTCCAAAACTGG	167, 588, 731
SSR-120	CGTTTAGCCACTAGCCTTGC	ACTCCTCGGATGAGGAGGAC	168, 079, 535
SSR-123	CAACTATAGCAAGCTGGCCC	GAGGCTCCAAATCAACGAAG	168, 238, 115
SSR-128	AAAGGGCCGAGTCTGTTTTT	CTGGGCATCATTCTTCAGGT	168, 512, 770
SSR-132	ACTCAGGCAGTTCAAGCCAT	ACGTTGGTGGATGACCTCTC	168, 745, 954
SSR-138	CTTGTGCCGTTCCAGATTTT	CCTGAACGGAGGAGACCATA	169, 073, 641
SSR-140	CCTTGGAGTTCAGCTTGGTC	CAAGAGCATTCTTGTTTGAGGA	169, 178, 272
SSR-146	GGGGTAGAAATTGTAATGCCC	CCAGCATGAGATGCAAGGTA	169, 484, 368
SSR-150	GATCCAATGGTCAAACCACC	GCGCATATTCAAGGTTCGAT	169, 724, 711
SSR-151	ATACTTGGTTCGAGCATCGG	ATGCTACCTGGTTGGGACAG	169, 792, 205
SSR-159	ACTCCTCGGATGAGGAGGAC	GAAGACCAGTGGCGTCTAGC	170, 262, 817


**Table 4:** The information of markers including in the predicted region.

Loci	Primer sequencing (5′–3′)	Position (bp)	LOD value
SNP-18	F: CGGTTACTACTACGGCAGCG	164, 677, 916	0.31
	R: CAGTTGTAGTAGGACGCCCC		
SSR-65	F: AGCCGATGGACAAAAATTGA	164, 685, 091	0.26
	R: TCGTCGTCTTCTGGACCTCT		
SSR-75	F: GCTGGGAAGAGGAAGAGGTT	165, 241, 204	37.20
	R: AAACAAGACGGGAACAAACG		
SNP-22	F: TTCCACCAGATCCTAAACGG	165, 243, 672	32.17
	R: GCAGATGCTACCAAGGCTTC		
SSR-78	F: ACACAAGAGGTGGGACAAGC	165,406, 106	45.23
	R: TGTACGTCTGGACCCTCTCC		
SSR-88	F: CCAAGGCACAAGAAGAGAGC	166,097, 976	0.47
	R: GCATGCATGGAAGAGGTACA		
SSR-92	F: AAAGACCAGTGGCGTTTAGC	166, 322, 914	0.36
	R: GGCTCGGATGAGTCTGAGTT		
SNP-25	F: CGTACCTCTTGACCTTGGGA	166, 721, 266	0.70
	R: AGCTACCACGTGCTGTCCTT		


### Candidate Genes for *GSR* Resistance

Based on the Maize reference genome (RefGen_V4, see text footnote 1), 33 genes were located in the predicted region (Supplementary Table S3). Based on gene annotation of the region, two genes, Zm00001d011953 (*Zm953*) and Zm00001d011972 (*Zm972*), were chosen as candidate genes. *Zm953* encodes an auxin response factor, and *Zm972* encodes a disease resistance protein. The physical location of *Zm953* (164, 991, 768) and *Zm972* (165, 428, 843) were near to SSR-78, which had the highest LOD value (**Table 4**).

**FIGURE 5:**
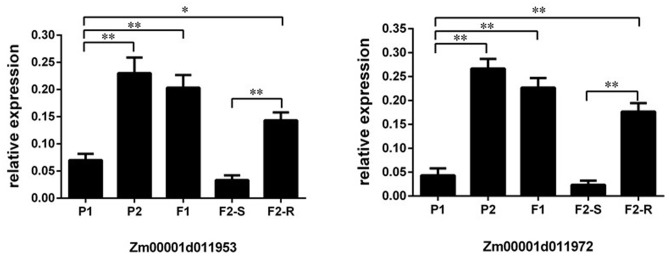
Relative expression of the candidate genes. ^∗∗^*P* < 0.01 and ^∗^*P* < 0.05, respectively.

In addition, the expression levels of *Zm953* and *Zm972* were investigated in two parental lines, the F_1_ plant, and the susceptible and resistant individuals in F_2_ by using qPCR. The results showed that the expression level of *Zm953* in the resistant plants from P2, F_1_, and F_2_-R was significantly higher than that in the susceptible plants from P1 and F_2_-S (**Figure 5**). The expression level of *Zm972* showed a similar result (**Figure 5**). Therefore, based on the gene annotation and results of expression analysis, we inferred that *Zm953* and *Zm972* may be the candidate genes for the major QTL conferring *GSR* resistance.

## Discussion

Maize stalk rot is a major and serious disease that reduces grain yield and quality (Yang et al., 2002a). In the past, several studies indicated a single dominant gene related to *GSR* resistance (Chen and Song, 1999; Yang et al., 2002b, 2004). However, other studies reported that *GSR* resistance was unlikely to be controlled by a single dominant gene, but was more likely to be a quantitative trait (Pè et al., 1993; Yang et al., 2010; Zhang et al., 2012). In this study, the F_2_ and BC_1_F_1_ populations derived from the cross “18327 × S72356” were used to analyze the inheritance of *GSR* resistance. The results obtained indicated that the *GSR* resistance was a quantitatively inherited trait.

Previous work has identified QTLs linked to *GSR* resistance through RAPD, RFLP, and SSR markers, and mapped on the chromosome 1, 3, 4, 5, 6, and 10, respectively (Pè et al., 1993; Yang et al., 2004, 2010; Zhang et al., 2012). No *GSR* resistance QTLs have been localized on chromosome 8. However, some resistant QTLs to *Gibberella* ear rot have been mapped on chromosome 8. Robertson-Hoyt et al. (2006) found a resistant QTL to *Gibberella* ear rot in the locus bin 8.03, explaining 10.7% of variation. Ding et al. (2008) located one QTL to *Gibberella* ear rot on bin 8.05, which accounted for 7% of the variation. In the present study, we identified and mapped one major genomic region harboring a *GSR* resistant QTL on chromosome 8, ranging from 161.001 to 170.6 Mb (contained in bin 8.06–8.08). We achieved this by studying the F_2_ population via the QTL-seq approach (Takagi et al., 2013a), which took advantage of the high-throughput genome re-sequencing and BSA. We named this major QTL as *Rgsr8.1*, which is located near the QTL conferring resistance to *Gibberella* ear rot detected by Ding et al. (2008). The result indicated that these two QTLs, *Rgsr8.1* (bin 8.06–8.08) and the QTL (bin 8.05) reported by Ding et al. (2008) were located on different regions of chromosome 8. Therefore, though these two QTLs are nearby, they are different loci. *Rgsr8.1* is a new *GSR* resistance QTL on chromosome 8.

Furthermore, based on the traditional QTL analysis using F_2_ and recombination test using BC_1_F_1_, we narrowed down the physical location of the resistance QTL *Rgsr8.1* to a 2.04-Mb interval on chromosome 8 that contributed 34.4% of the phenotype variation. As shown in Supplementary Table S3, 33 genes were located in this 2.04 Mb region. Among these genes, Zm00001d011953 (*Zm953*) and Zm00001d011972 (*Zm972*) were noteworthy based on the gene annotation of maize. The description of *Zm953* indicated that it is an auxin response factor, a transcription factor that binds specifically to the DNA sequence 5′-TGTCTC-3′ found in the auxin-responsive promoter elements. The annotation of *Zm972* is putative disease resistance protein RPP13-like protein. Recently, a transcriptome analysis of maize resistance to *F. graminearum* has been discussed (Liu et al., 2016), which posited that the *GSR* resistance is conferred by two QTLs, *qRfg1* (Yang et al., 2010) and *qRfg2* (Zhang et al., 2012). The results of the transcriptome analysis of *GSR* resistance indicated that *qRfg1* enhances *GSR* resistance through both constitutive and induced high expression of defense-related genes, and *qRfg2* confers the *GSR* resistance via relatively lower induction of auxin signaling (Liu et al., 2016). In addition, the physical position of *Zm953* and *Zm972* are 164, 991, 768 and 165, 428, 843 on chromosome 8. Thus, both are closed to the SSR-78 marker, which has the highest LOD value (**Table 4**). The expression analysis indicated that the expression levels of *Zm953* and *Zm972* in resistant plants were higher than in the susceptible plants (**Figure 5**). Therefore, we hypothesize that *Zm953* and *Zm972* are possible candidate genes for *Rgsr8.1*, and further experiments need to be done to further these observations.

Generally, the classical phenotypic selection of resistance to *GSR* is labor-intensive, time-consuming, and can be confounded by environmental factors. However, marker-assisted selection of disease resistance can be effectively deployed in crop breeding (Boyd et al., 2013). In this study, we developed an SSR marker (SSR-78) located at 165, 243, 672 on chromosome 8, and showed that it is tightly linked with the resistance genotype. Although the validation of SSR-78 has been verified using F_2_ and BC_1_F_1_ populations, more experiments are needed to confirm the results. Resistance plants could be selected at an early generation using the SSR-78 marker. The present results will be useful in maize breeding programs aimed at improving *GSR* resistance.

## Author Contributions

QC, JS, W-PD, and G-RY designed research. QC, YJ, JZ, and X-LX performed research. All authors analyzed the data. QC, L-YX, and G-RY wrote the paper.

## Conflict of Interest Statement

The authors declare that the research was conducted in the absence of any commercial or financial relationships that could be construed as a potential conflict of interest.

## Footnotes

1 maizesequence.org

2 http://pgrc.ipk-gatersleben.de/misa/

3 http://www.premierbiosoft.com/
