# Supplementary material for: Identification, Mapping, and Molecular Marker Development for Rgsr8.1: A New Quantitative Trait Locus Conferring Resistance to Gibberella Stalk Rot in Maize (Zea mays L.)
Source: Front Plant Sci. 2017 Aug 3;8:1355. doi: 10.3389/fpls.2017.01355 (PMC5540892; doi:10.3389/fpls.2017.01355)
Supplement: Supplementary file 1 [file Table_1.DOCX]

Table S1 The information of SNP markers

| Loci | Forward primer (5'-3') | Reverse primer (5'-3') | Position (bp) |
| --- | --- | --- | --- |
| SNP-1 | ATTTTAAGCCAAGCGGGATT | GCAACAGAGCAATTTCGTGA | 161, 004, 365 |
| SNP-2 | TTCCCGTGCATTCTCCTTAC | CAAAGCAAACCACACCATTG | 161, 299, 430 |
| SNP-3 | CGGAATATCTCGCAACAGGT | CTCTTCCTGGAGTCCTCGG | 161, 466, 693 |
| SNP-4 | GATGTGATGCTGCTGGAAGA | TCAAACCCTCTGCTCGCTAT | 161, 666, 100 |
| SNP-5 | GTCATGGAGATGGAGGTCGT | ACGCTGCCTACCTCCGCT | 162, 145, 869 |
| SNP-6 | CTGGTTCCTGGATAGGCTCA | GGTGCAGCACGTGGATTTAT | 162, 303, 993 |
| SNP-7 | CGTGCTACAACGTGACCATC | GATTTCAGGCCGTACAGAGC | 162, 541, 729 |
| SNP-8 | CGATCTTGACTTGACGACGA | CGTTGGTCAGCTCCTTCTTC | 162, 652, 623 |
| SNP-9 | TTGGCAAGAATGGAGGTAGG | TCACTGAATCTCTTCCGGCT | 162, 804, 787 |
| SNP-10 | GTCTTGGTTGGCATTCCACT | GTTTGAAAGCCCGTGGACTA | 162, 949, 082 |
| SNP-11 | CAGGGTATGGTCAGGCAACT | TAGACCGTGGCTAATGGCTT | 163, 348, 943 |
| SNP-12 | GAAGAACTTGCCGACGACAC | ACAGACGGAGTGCTTCCAGT | 163, 865, 024 |
| SNP-13 | AACAAATGCACAAATGGCAA | TTTTCGCCATTTCAGTTTCC | 163, 940, 686 |
| SNP-14 | ATTGAAGCCGTCGAACATCT | AACGCCAGTTACCACTCACC | 164, 115, 370 |
| SNP-15 | GAATTGAGATGGGGGAGTGA | GTGCTGTACGCCTGTACCCT | 164, 253, 779 |
| SNP-16 | AGGGACTTCAAGACGAGCAA | ACGTACATTTCATCCAGGGC | 164, 360, 441 |
| SNP-17 | ATATGCCCATGCTCTCCAAG | AGCCTGTGGGTTTCAAAATG | 164, 543, 886 |
| SNP-18 | CGGTTACTACTACGGCAGCG | CAGTTGTAGTAGGACGCCCC | 164, 677, 916 |
| SNP-19 | CGTTTCGTACGTGTGTCTGG | CCATCACGTACACCAGCATC | 164, 802, 059 |
| SNP-20 | CGTCTTGCAGAATGACCTGA | TCGGGACGACTTTATTCTCG | 164, 995, 289 |
| SNP-21 | TTCCACCAGATCCTAAACGG | GCAGATGCTACCAAGGCTTC | 165, 243, 672 |
| SNP-22 | TGCAGAAGCTAAAGCTCGGT | TTGTGGTAATCCTGGCCTTC | 165, 429, 540 |
| SNP-23 | TGATGGTCATGTGCTCCTGT | CCCATCCTCTGGTGTCATCT | 165, 792, 665 |
| SNP-24 | CCCAGCTTCAGGTCCAGC | GGCTGTAAGCCTGTAGCCAT | 166, 517, 287 |
| SNP-25 | CGTACCTCTTGACCTTGGGA | AGCTACCACGTGCTGTCCTT | 166, 721, 266 |
| SNP-26 | AAGAAACTGCTCCTTGGGGT | ATTCCTTCTCCGTGGGATCT | 166, 791, 988 |
| SNP-27 | CCAGTCGACCATAGCTGTCA | ACCAAGGTTCGTTCAAATGC | 166, 901, 685 |
| SNP-28 | AGGATGAGGAGGAGGAGAGC | CGAGGATCTCCTGGATGAAG | 167, 199, 003 |
| SNP-29 | CTGATGGCAGGGTTCAAAAT | AAAGGTGGCTTTGAGCTTGA | 167, 474, 984 |
| SNP-30 | AAGGCCAACTGCAGAAGAAG | GGAGGAAATCTAAGACCGCC | 167, 672, 380 |
| SNP-31 | CCAACGCGTCGTTACAGTTA | CACTCACCTGCTCCTGCC | 167, 839, 590 |
| SNP-32 | TCTCTCAGCAAAGGACGGTT | GGGGGATGACCTTGAGTTTT | 168, 227, 821 |
| SNP-33 | CCTAATAGTTTCCCCGGCTT | TATCTTCTCAGAGCAGCGCA | 168, 610, 967 |
| SNP-34 | GTAGGAGACGGAAATGCTGG | GTCTCAATCGCATCTGCAAC | 168, 866, 870 |
| SNP-35 | GTAGGAGACGGAAATGCTGG | GTCTCAATCGCATCTGCAAC | 168, 866, 870 |
| SNP-36 | CCAAACCAATGCAACATCAG | TTGCCACGATATGGTCTTGA | 169, 185, 032 |
| SNP-37 | GGCGCTATGGACTACCATGT | TCGCGTCAATAGATCAAACG | 169, 443, 543 |
| SNP-38 | ATGCAAGCAATGATGGAACA | AGTGTGGTGGTGAAAAAGGC | 169, 612, 381 |
| SNP-39 | CACAAAATTCGGAGTCCACC | CTGTTGCGTCGAGTAGGTCA | 169, 777, 942 |
| SNP-40 | TACCAGGAAGCCACAATTCC | GCTGGAGCTATGTGGTGGAC | 169, 944, 226 |
| SNP-41 | TCAGCTCGCTCACATTTGTC | AACAATCTAGGATCGCGGAA | 170, 084, 205 |
| SNP-42 | CACACCTGGAAGAACCCGT | AGCCCCAGCATTGACCTC | 170, 118, 171 |
| SNP-43 | TGACAGGAGAGAATTTGGGG | CAAGCTCATTCCAAGCATCA | 170, 396, 935 |
| SNP-45 | GTCTTCGCTTCGGTTGAGAG | TGCTGATGGGGAAGTAGACC | 170, 534, 556 |
