# Supplementary material for: Identification, Mapping, and Molecular Marker Development for Rgsr8.1: A New Quantitative Trait Locus Conferring Resistance to Gibberella Stalk Rot in Maize (Zea mays L.)
Source: Front Plant Sci. 2017 Aug 3;8:1355. doi: 10.3389/fpls.2017.01355 (PMC5540892; doi:10.3389/fpls.2017.01355)
Supplement: Supplementary file 2 [file Table_2.DOCX]

Table S2 The information of SNP markers

| Loci | Forward Primer (5'-3') | Reverse Primer (5'-3') | Position (bp) |
| --- | --- | --- | --- |
| SSR-1 | ATCAAATTTGTTGCCTCGCT | AAATTGGCTCCTCCCTGTTT | 161, 018, 018 |
| SSR-2 | CACGTTAGAGAGACGGCACA | CCAGTTAAGCCACTGCAACA | 161, 078, 201 |
| SSR-3 | ATGCTGAGGCGCAACTCTAT | GGACTGGTGAGGTTTCCGTA | 161, 174, 965 |
| SSR-4 | GACGGAACCAGCTGAGAAAA | GGAGGATTGCAGAGAGATCG | 161, 253, 366 |
| SSR-5 | AACGACATGGCCTTACCTTG | CTACACCTCTGTGCCACCAA | 161, 304, 360 |
| SSR-6 | ACTCCTCGGATGAGGAGGAC | AAGACCAGTGGCATCTAGCC | 161, 350, 979 |
| SSR-7 | GAGGAGAGGGAGGAAGGAGA | TTTTTCCAAAAATCCTCCCG | 177, 502, 201 |
| SSR-8 | ATCTGTGGTGGTGTCACCTT | GAATTCACTGCTCCATGTGC | 161, 447, 742 |
| SSR-9 | CAACGAGCTAGGTAGCAGGC | AGCCTCAATGATGTTGCTCC | 161, 507, 249 |
| SSR-10 | CATGAGGGCTGGATACTTGG | TTCGTTGGTACATTGATGTGG | 161, 555, 503 |
| SSR-11 | AAGCAGCATTGGTAGTCGGT | CCACAACCTCAACCTCCACT | 161, 722, 619 |
| SSR-12 | GACCGTTGTCGTTGTCATTG | TTCCAGTTTTGCTCTACACTCAA | 161, 764, 284 |
| SSR-13 | AAAGGTACGCCACGTCATTC | TGACCAGTAGTGGGTGTGGA | 161, 872, 013 |
| SSR-14 | CGACGAAATAAATTCCCCAA | CTTCACCATCCGGTTCAGTT | 161, 930, 664 |
| SSR-15 | GCTCTGGCTCCTCTCTGCTA | TGCAGTAGGGGGTTACAAGC | 161, 973, 272 |
| SSR-16 | AGTCAACTTTGGGTTGCTGG | GTGCAAATGTGGCATTGAAG | 162, 020, 659 |
| SSR-17 | CTCTCTCCCCTCTGGTGTTG | TGCTGCTGCCATTTCTCATA | 162, 065, 339 |
| SSR-18 | CCCATGGGAAGTTGAACCTA | CAAGCCCCCTTATGATCTTG | 162, 113, 979 |
| SSR-19 | TGGATTTAAAGGCCTGATGG | TGGATCGATGAAAACGATGA | 162, 158, 649 |
| SSR-20 | CTATTGGTTGGCCATTTGCT | TGGGAATCTCCACTTCTTGG | 162, 216, 817 |
| SSR-21 | GACATCACATGAACAGGGCA | GCTTGCAATAGGGGGTTACA | 162, 263, 051 |
| SSR-22 | ATTAGGTTTTAGCAGGGGCG | ATCCTAGAGGGAGCGTCACC | 162, 319, 997 |
| SSR-23 | AAGTAACAGGACACAGCGGG | CCGTAAGGCACACTGCACTA | 162, 368, 778 |
| SSR-24 | ACCGTGATCTTTGGAAGTCG | GCATTCCGATAGGGATTACG | 162, 415, 963 |
| SSR-25 | ATAGACGTCCGGATGTGGTC | AAGGCCTGATCACATAATCCA | 162, 461, 145 |
| SSR-26 | CATAGCTAGCAAGCACGCAC | CTGAACCCTCCATTTTCTGC | 162, 540, 029 |
| SSR-27 | CGGAAGAGAAGTCGATCAGG | TTCTTCTTCGTCGTCGTCCT | 162, 574, 600 |
| SSR-28 | TAGTTGGGTCCAGATCCAGG | AACAGGTCCGTCCAGTTCAC | 162, 639, 717 |
| SSR-29 | GATTTCGTAGCAACGCACG | CATTGAGATGGGTGACATGG | 162, 680, 266 |
| SSR-30 | CTCCTCCAGAGGTCAACGAG | CTGCTCCCGATCATCAAAAT | 162, 714, 637 |
| SSR-31 | TCTGAGGTTTTCATACGGGG | CCAAACAACACCTTGCTGTG | 162, 779, 032 |
| SSR-32 | CTGCAACTGAGATGGTCCAA | GGGTATCACGTCGTCTTCGT | 162, 828, 637 |
| SSR-33 | AGTTGTTCGTCAACACGCAG | CTCGTAAAAGAGGTGGTCGC | 162, 875, 404 |
| SSR-34 | TGTTTGGTTTGTGGAATGGA | CCGCTAAACTCGCACTTAGG | 162, 924, 552 |
| SSR-35 | TCTCTATCTCTCTCCCTCTCCC | AGAGAGAGGAGGGCACGC | 162, 959, 644 |
| SSR-36 | AGTCCACGAAACAGGGAAAA | TTCATGGCTAAACAAAGGCA | 163, 010, 657 |
| SSR-37 | TGTACACCTGGACCCTCTCC | TAAAATTGTCGCTGCGTGTC | 163, 080, 386 |
| SSR-38 | GCTTGCAGTAGGGGGTTACA | TAAAAGGAAGGTCCAGGGCT | 163, 149, 402 |
| SSR-39 | ACAACTGGCTGTGCAAAGTG | TGCTGCTGTCAAGTTCCATC | 163, 198, 928 |
| SSR-40 | ATGGACAAGCCATTAGCCAC | TCAGCGGACGTGACTGTTTA | 163, 222, 584 |
| SSR-41 | CAACTGGCTGTGCAAAGTGT | GACCCTTTCTGGATGGTTCA | 163, 319, 000 |
| SSR-42 | TGCATCATGCTGGGATTTTA | CATTGAACCCAAAAATATGGA | 163, 391, 268 |
| SSR-43 | TCAAGCTCTGATTTTCCCAA | CATTCACGATGTTCCTTGGAC | 163, 442, 237 |
| SSR-44 | AGAGAGAGGGGAAAGCGAGT | AAAGGAAAGAAGCCCACTCC | 163, 495, 693 |
| SSR-45 | CATTCGGTCGGATATTGCTT | GTCATTGATCTTGCACCTGG | 163, 567, 953 |
| SSR-46 | AGCACCCTCCTCGGAGTATT | CTCCCCTAACACTGTAGCCG | 163, 633, 472 |
| SSR-47 | GGTCAACATGGTCATCTCCC | AAACACTTGTTCCCATTGCC | 163, 689, 206 |
| SSR-48 | AACATTCTGCACCTGCCTCT | AACACACAGAACAGGGGACC | 163, 732, 434 |
| SSR-49 | AGATGCACAATTGCTTGCTG | GCAGTGCCCTCAGTTAGACC | 163, 762, 731 |
| SSR-50 | AGCTTTTCACCTCCACGCTA | TAGCTCCAACACGTACACGG | 163, 819, 019 |
| SSR-51 | TTGTTCCTTTCTTCCATGCC | GGTGTATGCGTAATGCGTTG | 163, 870, 956 |
| SSR-52 | TCCGATGATCACAGTCTCCA | GTGCGTGTCGTAGGTTTCAA | 163, 895, 757 |
| SSR-53 | AAGCCGATTCACTGAGCCTA | TTGTAGAGCTGCACCACGTC | 163, 949, 635 |
| SSR-54 | TGTTGGCAGTTCGTATCAGC | TCTCTGTAGGCTCCAAATCCA | 164, 017, 140 |
| SSR-55 | ATCGGATTCGGATCCTCTCT | AATGACCAGCGACCAAAAAC | 164, 049, 484 |
| SSR-56 | ATTTCCCGTTCTGCTGTTGT | GACCGAACACGGTAAGCAAT | 164, 098, 887 |
| SSR-57 | GTTGTCGCCAGGAAAGACAT | CATGTTCAACGAAGTGGTGG | 164, 161, 814 |
| SSR-58 | GCCCAAAGATGACAAGCCTA | GTTGTGGTCGTCGTCCTTTT | 164, 209, 325 |
| SSR-59 | CGACCTGGATAAGAAGCAGC | AGCATGCAATGTCCAGTTGA | 164, 257, 593 |
| SSR-60 | GCGCTTATCGTTCAGGATTC | AATAAGCCAAAAAGGGTCCG | 164, 310, 201 |
| SSR-61 | ATGGTGGTGCCTCAACTCTC | TTGCTCGTCTTGAAGTCCCT | 164, 360, 267 |
| SSR-62 | TTCATCAACCAGGAAGGACA | AAACATCATTAACCGTCGCA | 164, 407, 505 |
| SSR-63 | GTTGAACAAAGATGGTGGGC | AACCTGAGGAGCCAGAACCT | 164, 459, 430 |
| SSR-64 | CAGTGGTTGACATGCTGCTT | CCAGCTCACCTGCTATCTCC | 164, 601, 954 |
| SSR-65 | AGCCGATGGACAAAAATTGA | TCGTCGTCTTCTGGACCTCT | 164, 685, 091 |
| SSR-66 | AAAAAGGTGCGCCACACTAC | CTGATCAGTAAAGGGCTCGG | 164, 738, 774 |
| SSR-67 | ACGGATACATCACCTGAGGC | TGTAAACATTTACGCGCAGC | 164, 785, 799 |
| SSR-68 | CAGTTCGCAAATAGACCAGATG | TCATGAGCATTTGTTCCTTCA | 164, 825, 611 |
| SSR-69 | CAGGTGGATAAAGAAACCAAACA | TTTCTGTTCTTTGGGGGTTG | 164, 878, 776 |
| SSR-70 | ACGTTGGTGGATGACCTCTC | ACTCAGGCAGTTCAAGCCAT | 164, 937,112 |
| SSR-71 | CATCCAGCTTGTGACGATTG | TTTAGAGGTAACGGTTGCGG | 164, 999, 715 |
| SSR-72 | CTCTCCGTCAGAGGACAAGG | TGGTGCTGGTGGTGTGTACT | 165, 049, 553 |
| SSR-73 | AGTTGGTCGGCTGAGAGAAA | AACAAGTAATGTGTGTGCATTTCA | 165, 120, 828 |
| SRR-74 | CATGAATTCAGCACGCTTGT | GATGTGTGGTTGCATTCTGC | 165, 170, 058 |
| SSR-75 | GCTGGGAAGAGGAAGAGGTT | AAACAAGACGGGAACAAACG | 165, 241, 204 |
| SSR-76 | GTAGTACCTGAAGCTGGGCG | GTACAGCGAGGCGTCCAC | 165, 310, 272 |
| SSR-77 | GGGACCACCACGAGAAGATA | GGATCATCACATAGCCCGAT | 165, 340, 004 |
| SSR-78 | ACACAAGAGGTGGGACAAGC | TGTACGTCTGGACCCTCTCC | 165, 406, 106 |
| SSR-79 | TGGCACTATGGTCCATCTGA | CTTCCTCCCATGCCTTCC | 165, 481, 525 |
| SSR-80 | ACCCGTTGGCTCCTACTTCT | GAAGCTGTCGCACTTGTTGA | 165, 501, 654 |
| SSR-81 | ACGACATACAGGTGCCATCA | AGAAGGAGGTGGCGCTCT | 165, 566, 698 |
| SSR-82 | GACGAAGCAGCTGACAAGC | GCCTCCCTCTCTCTAGCAAA | 165, 671, 084 |
| SSR-83 | CAACTCCAACAAGAGCAGCA | CTGCAACAATTGCTCAAGGA | 165, 743, 501 |
| SSR-84 | AAGGATTGGAGAAGGGGCTA | AATACAAGTTTTGCGCCACC | 165, 881, 263 |
| SSR-85 | TGTCTTCAGGCTACTTAAAGGAAAA | CAACAGCTAGGTTTCAACGGA | 165, 942, 745 |
| SSR-86 | ACACGTGTAGCGTGAGGATG | TTGGGACTAGGGCATGGTAG | 165, 999, 276 |
| SSR-87 | TGGATCTTTTATGGGCTTGG | CAGACGTACGCGGTTAGTGA | 166, 048, 867 |
| SSR-88 | CCAAGGCACAAGAAGAGAGC | GCATGCATGGAAGAGGTACA | 166, 097, 976 |
| SSR-89 | GTTCACATTTTCGGGATGCT | AACGAAGGATGGATGGACAA | 166, 142, 532 |
| SSR-90 | TTAGGGCGCTTGTAGCATTC | CGCGACCCAAGAGATAAGAG | 166, 199, 308 |
| SSR-91 | ACGACGCAGAGAGAGGAAAG | AGAAAAGGAGAAAGCAGCCC | 166, 265, 043 |
| SSR-92 | AAAGACCAGTGGCGTTTAGC | GGCTCGGATGAGTCTGAGTT | 166, 322, 914 |
| SSR-93 | TTCCTGTTCACTTGTGCCTG | ATTCCTCAAGCAAGGAAGCA | 166, 369, 609 |
| SSR-94 | AAGCCATTTCATGGCAACTC | ATTATTTGGACGAACGGACG | 166, 428, 954 |
| SSR-95 | GACCTGCCGCGAGTAGTG | TCTCCCTCTTCCTCTCTCCC | 166, 461, 930 |
| SSR-96 | CGTATTTGTCTGCAAAGCGA | CTAAACCCTGAAACGCCAAA | 166, 520, 112 |
| SRR-97 | AAGCATTCTTGGCACTCGAT | ATGTGGTTTGCCTTACCCTG | 166, 555, 384 |
| SSR-98 | TCATGATGGAATGCGAAAAA | TCAAGCCGAGTTGGATCTTT | 166, 614, 499 |
| SSR-99 | GACTTCCACCTCTCTCACGC | TCTCCACTTGGCCCCTAATA | 166, 649, 811 |
| SSR-100 | GCACCTATATGAAGCCCAGG | CCCCAAACTTCCAAAAAGTG | 166, 780, 249 |
| SSR-101 | TAGAAACTCGTTTCGGTCGG | CCGCACTGTTTGGACATCTA | 166, 814, 964 |
| SSR-102 | AGTGAGCCTTGAGCACCATAG | AATTTCCATTGATTCGGTGC | 166, 862, 760 |
| SSR-103 | CAGGTCTACCCACTTGCACA | TTAGAAACGACGGCAAAAGC | 166, 919, 170 |
| SSR-104 | GCAGCACTAGAAATCCCAGC | CTCATGCCTCGTTTTTGGAT | 166, 970, 190 |
| SSR-105 | CGGCAAATCATCAAGCCTAT | AATTAGCCGTTGAAGCGAAA | 167, 025, 465 |
| SSR-106 | GGATAAAAGCATCGGGGTTT | GTCTAGTGCTCTATCGGCGG | 167, 096, 554 |
| SSR-107 | TGCTTGGTTGCTCTGATTTG | CACGTTTCTCAGGCAGTTCA | 167, 159, 153 |
| SSR-108 | TGCTGGTGCTGCTATCAATC | AAATTTTCCGGAACAGGGAC | 167, 203, 179 |
| SSR-109 | TTTGTGTACGTACCGGGTGA | TGAGCGACACCAGCATAGAC | 167, 245, 500 |
| SSR-110 | CACCTATGCGCAGAGTTTGA | GGCATCGTTTTCTTTTCCAA | 167, 308, 770 |
| SSR-111 | TCGAGCTCCCATTTATCAGC | TCTGGTCTGGGCTTCCTCTA | 167, 530, 840 |
| SSR-112 | GCTCTGCTTCTCACTAGCGG | ACAGAGCCTTCCAAAACTGG | 167, 588, 731 |
| SSR-113 | GCCTCATCTGAAAGCTGGAC | CAGCCGTGTGGTAAGGATTT | 167, 602, 697 |
| SSR-114 | TTTTGGTCGGCTCAAATCTT | TACAACCCATCCTCGACACA | 167, 715, 493 |
| SSR-115 | ACTCGCGTGCCATTATTAGG | AAACGCACTAAGAGCATGGC | 167, 767, 528 |
| SSR-116 | GGAAAACACGAGGGAACAAA | TCCGATTTTAGGGGATGAGA | 167, 839, 915 |
| SSR-117 | CTTTTCCTTGCCAAAACAGG | AGGAGCACCACCAAGTGTTT | 167, 847, 482 |
| SSR-118 | GCTAATGTGCCTCCGAGCTA | CACTCCACCTAAGAGCAGGC | 167, 974, 131 |
| SSR-119 | CAATGATCAAGGTTGCGATG | GCAGTGTGAATCTCCGGTTT | 168, 038, 343 |
| SSR-120 | CGTTTAGCCACTAGCCTTGC | ACTCCTCGGATGAGGAGGAC | 168, 079, 535 |
| SSR-121 | TCGAATGATCGAATGAACGA | AAGCCATTTCATGGCAACTC | 168, 133, 270 |
| SSR-122 | AGCAGGAGAGGGATGGACTC | AACTACTTCAGCGGAGGGGT | 168, 189, 112 |
| SSR-123 | CAACTATAGCAAGCTGGCCC | GAGGCTCCAAATCAACGAAG | 168, 238, 115 |
| SSR-124 | GAGGTGATGGTCGTAGGCAT | TTCGAAGGTTGCAAAGAAGG | 168, 290, 235 |
| SSR-125 | CAACACCCAATGATTCCCTC | AGTTGAATGGGACTCGGATG | 168, 340, 096 |
| SSR-126 | TCCACCTTGTCAGCAAACTG | GTAAGACCTCCCCCTGAAGC | 168, 376, 194 |
| SSR-127 | GCCATCACTGAAGCAACTGA | CTCTAATCGCGCATTTGGTC | 168, 443, 154 |
| SSR-128 | AAAGGGCCGAGTCTGTTTTT | CTGGGCATCATTCTTCAGGT | 168, 512, 770 |
| SSR-129 | CTAGCCCGGCCCATATTATT | GAGTCACGGACCATTCCCT | 168, 563, 113 |
| SSR-130 | ATTGGTTGGGGTTCGTATCA | ACAACACCGACCTCTTCGAC | 168, 648, 660 |
| SSR-131 | CCTAATAGACCGAATGCCCA | ATACGTGTCCACTGGTCGCT | 168, 709, 034 |
| SSR-132 | ACTCAGGCAGTTCAAGCCAT | ACGTTGGTGGATGACCTCTC | 168, 745, 954 |
| SSR-133 | GGGCTTAATCCAATTTCTAGTCA | CCAACCTCGGTATAACCCTTC | 168, 796, 541 |
| SSR-134 | TGCAGAGAGCCAGAGAGACA | CCGAGTCGAGCTCGTACAGT | 168, 867, 255 |
| SSR-135 | CATTGGTGCTCTCTTTTCCA | CATGTGGGCATTTTGATGAG | 168, 928, 072 |
| SSR-136 | GTTGCCAGTTGGAAAGAAGG | ACGTCCTGGAAGAAGCAAGA | 168, 996, 960 |
| SSR-137 | CTTTAGCAGCGGCGATTTAC | ATCTTTCGCAGGAGCTTCAA | 169, 048, 456 |
| SSR-138 | CTTGTGCCGTTCCAGATTTT | CCTGAACGGAGGAGACCATA | 169, 073, 641 |
| SSR-139 | GAATTCACTGCTCCATGTGC | ATCTGTGGTGGTGTCACCTT | 169, 129, 707 |
| SSR-140 | CCTTGGAGTTCAGCTTGGTC | CAAGAGCATTCTTGTTTGAGGA | 169, 178, 272 |
| SSR-141 | GAGCTCGTAAATGAGCCGAG | CCTTTAGTTTCCCACATGCC | 169, 202, 148 |
| SSR-142 | CTGTCATCACACTGAGCGGT | TTCTGATGCAGACTCAAGCG | 169, 255, 461 |
| SSR-143 | CCAAAAGGTTTGTCTTCCCA | GAGCTTCGCTTTGCTCTCTC | 169, 300, 355 |
| SSR-144 | ACGGGATCCGAGTAAGGAAC | ATGATCATGCGGAGAACACA | 169, 361, 040 |
| SSR-145 | GGGTTTATCTTCGCGGGTAT | GGGTGTAGGGTTTGCTGAGA | 169, 522, 695 |
| SSR-146 | GGGGTAGAAATTGTAATGCCC | CCAGCATGAGATGCAAGGTA | 169, 484, 368 |
| SSR-147 | CGAGCGATCAAGTCGAAGA | CTAGCGCCTGTCTTTCCATC | 169, 540, 996 |
| SSR-148 | TTCTAAACCCTCGTCGTTCG | TATGAACACGGACAGATGCG | 169, 574, 512 |
| SSR-149 | GACGAAGCCATCCGCTATAC | CTTCCTTCTGTGCGAAAAGC | 169, 621, 452 |
| SSR-150 | GATCCAATGGTCAAACCACC | GCGCATATTCAAGGTTCGAT | 169, 724, 711 |
| SSR-151 | ATACTTGGTTCGAGCATCGG | ATGCTACCTGGTTGGGACAG | 169, 792, 205 |
| SSR-152 | GAGCCATTCGATGCTACCTC | GCCATCCATGGAATTCTTGT | 169, 860, 584 |
| SSR-153 | CAACTCTGTTCCTCTGTGCG | ACCTCAATCAGGTTCGGTTG | 169, 934, 369 |
| SSR-154 | CGATCGGATCAAGAGGATGT | CTCTTAGGCGAAGCGCTAGT | 170, 005, 118 |
| SSR-155 | TTTGCCATCAAACTCTGGAA | CCTCGTTGTTGTTTGCTCCT | 170, 071, 090 |
| SSR-156 | GAGGTCAATGCTGGGGCT | CCTGTCCTCCCCTGTTACAA | 170, 118, 380 |
| SSR-157 | GCAGAGGTAACGGCAGATGT | CCCCCTAAGAGAACCTGGAA | 170, 164, 522 |
| SSR-158 | GACAAGAGCGCAAATGAACA | TTGGCATCCAAGTTTTCAGA | 170, 209, 573 |
| SSR-159 | ACTCCTCGGATGAGGAGGAC | GAAGACCAGTGGCGTCTAGC | 170, 262, 817 |
| SSR-160 | GCGCACACCAAAAGAGTGTA | CGGGGCTTCCATTAGTTAGA | 170, 318, 502 |
| SSR-161 | AGTAAACATTCGGCCCTGTG | GCCTTCAGAGATTTGGTTGTG | 170, 365, 606 |
| SSR-162 | GAAGGACTCATCAATCGGGA | TCGTCATGTTCAGCTCCTTG | 170, 419, 070 |
| SSR-163 | AGAGAGGCGGATAGGGAGAG | ATCAACGGAGACCACAGACC | 170, 466, 551 |
| SSR-164 | CTTCGAGAGTTGGAGCCCTA | TGCTCGTCTTGCTCGTAGTC | 170, 532, 933 |
| SSR-165 | TGAAGCCACACACAACAACA | AGTTGCAAGCCAGTGTGATG | 170, 599, 915 |
