# Supplementary material for: Identification, Mapping, and Molecular Marker Development for Rgsr8.1: A New Quantitative Trait Locus Conferring Resistance to Gibberella Stalk Rot in Maize (Zea mays L.)
Source: Front Plant Sci. 2017 Aug 3;8:1355. doi: 10.3389/fpls.2017.01355 (PMC5540892; doi:10.3389/fpls.2017.01355)
Supplement: Supplementary file 3 [file Table_3.DOCX]

Table S3 Predicted genes within the region from 164.678 to 166.721 on chromosome 8.

| Gene ID | Position (bp) | annotation |
| --- | --- | --- |
| Zm00001d011924 | 164, 685, 409 | PREDICTED: protein LURP-one-related 11-like [Zea mays] |
| Zm00001d011927 | 164, 786, 383 | PREDICTED: tafazzin homolog [Zea mays] |
| Zm00001d011928 | 164, 791, 252 | PREDICTED: heparan-alpha-glucosaminide N-acetyltransferase-like [Oryza brachyantha] |
| Zm00001d011930 | 164, 803, 203 | tropinone reductase 2 [Zea mays] |
| Zm00001d011932 | 164, 812, 818 | cytochrome P450 89A2-like precursor [Zea mays] |
| Zm00001d011938 | 164, 854, 662 | Os02g0462000, partial [Oryza sativa Japonica Group] |
| Zm00001d011940 | 164, 858, 334 | membrane protein LEM1 [Zea mays] |
| Zm00001d011943 | 164, 885, 894 | long chain base biosynthesis protein 2d [Zea mays] |
| Zm00001d011944 | 164, 891, 440 | BnaA10g20040D [Brassica napus] |
| Zm00001d011945 | 164, 894, 914 | PREDICTED: unconventional prefoldin RPB5 interactor-like isoform X3 [Zea mays] |
| Zm00001d011952 | 164, 981, 728 | PREDICTED: tRNA (adenine(58)-N(1))-methyltransferase non-catalytic subunit trm6 isoform X2 [Zea mays] |
| Zm00001d0119533 | 164, 991, 768 | PREDICTED: auxin response factor 4 [Zea mays] |
| Zm00001d011955 | 165, 136, 558 | PREDICTED: uncharacterized protein LOC103636331 isoform X2 [Zea mays] |
| Zm00001d011956 | 165, 140, 731 | PREDICTED: LOC542089 isoform X2 [Zea mays] |
| Zm00001d011958 | 165, 203, 758 | glutathione S-transferase,-like protein [Zea mays] |
| Zm00001d011959 | 165, 243, 021 | secondary cell wall-related glycosyltransferase family 47 precursor [Zea mays] |
| Zm00001d011963 | 165, 267, 196 | ATP-dependent RNA helicase DHX8 [Zea mays] |
| Zm00001d011967 | 165, 308, 568 | PREDICTED: probable serine/threonine-protein kinase At1g54610 [Zea mays] |
| Zm00001d011968 | 165, 309, 680 | PREDICTED: matrix-remodeling-associated protein 7-like [Brachypodium distachyon] |
| Zm00001d011969 | 165, 321, 330 | NAC domain-containing protein 68 [Zea mays] |
| Zm00001d011971 | 165, 345, 673 | delta3,5-delta2,4-dienoyl-CoA isomerase [Zea mays] |
| Zm00001d011972 | 165, 428, 843 | disease resistance protein RPP13-like [Zea mays] |
| Zm00001d011975 | 165, 490, 993 | PREDICTED: UPF0400 protein C337.03-like [Setaria italica] |
| Zm00001d011982 | 165, 792, 570 | dynamin-related protein 1A [Zea mays] |
| Zm00001d012005 | 166, 370, 584 | histidine kinase 2 [Zea mays] |
| Zm00001d012007 | 166, 419, 740 | PREDICTED: CBS domain-containing protein CBSCBSPB1-like [Brachypodium distachyon] |
| Zm00001d012010 | 166, 461, 949 | uncharacterized protein LOC100277040 [Zea mays] |
| Zm00001d012014 | 166, 472, 452 | Os08g0290200 [Oryza sativa Japonica Group] |
| Zm00001d012015 | 166, 514, 341 | squamosa promoter-binding protein-like (SBP domain) transcription factor family protein, partial [Zea mays] |
| Zm00001d012016 | 166, 534, 747 | PREDICTED: F-box protein At1g67340 [Zea mays] |
| Zm00001d012017 | 166, 556, 251 | mitochondrial import receptor subunit TOM20 [Zea mays] |
| Zm00001d012021 | 166, 634, 583 | PREDICTED: uncharacterized protein At1g28695-like [Zea mays] |
| Zm00001d012023 | 166, 715, 349 | regulatory protein [Zea mays] |
